# Weighted nearest neighbours-based control group selection method for observational studies

**DOI:** 10.1371/journal.pone.0236531

**Published:** 2020-07-23

**Authors:** Szabolcs Szekér, Ágnes Vathy-Fogarassy

**Affiliations:** 1 Department of Computer Science and Systems Technology, University of Pannonia, Veszprém, Hungary; 2 Healthcare Business Analytics Research and Development Centre, University of Pannonia, Veszprém, Hungary; Roswell Park Cancer Institute, UNITED STATES

## Abstract

Although in observational studies, propensity score matching is the most widely used balancing method, it has received much criticism. The main drawback of this method is that the individuals of the case and control groups are paired in the compressed one-dimensional space of propensity scores. In this paper, such a novel multivariate weighted *k*-nearest neighbours-based control group selection method is proposed which can eliminate this disadvantage of propensity score matching. The proposed method pairs the elements of the case and control groups in the original vector space of the covariates and the dissimilarities of the individuals are calculated as the weighted distances of the subjects. The weight factors are calculated from a logistic regression model fitted on the status of treatment assignment. The efficiency of the proposed method was evaluated by Monte Carlo simulations on different datasets. Experimental results show that the proposed Weighted Nearest Neighbours Control Group Selection with Error Minimization method is able to select a more balanced control group than the most widely applied greedy form of the propensity score matching method, especially for individuals characterized with few descriptive features.

## Introduction

Case-control studies are widely applied risk analysis methods mainly used in healthcare and social sciences. In retrospective medical studies, the case group typically contains diseased patients, while the control group includes non-diseased individuals, and the study aims to evaluate those risk factors that potentially led to disease occurrence. In the case of prospective studies, typically the effect of a treatment, intervention or other factor is evaluated by comparing the outcome variables of the case (treated) and control (untreated) groups. The popularity of these studies arises from their relatively inexpensive nature, however, the degree of their evidence is lower than randomized trials. The reliability of these studies can be increased by (1) increasing the number of cases included in the study, (2) performing thorough data preparation and data cleaning activities and (3) selecting a proper control group for the case group. The latter highlights the fact that the primary aim of case-control studies is to compare individuals in the case and control groups. A prerequisite of carrying out appropriate analyses requires that the case and control groups are similar on covariates that predict group membership (e.g. treatment assignment) and affect the examined output. However, fulfilling these requirements is not a trivial task. Many articles have highlighted the importance of the proper implementation of a control group selection method and the effect of unbalanced control groups on the result of analyses [1–5].

Nowadays, propensity score matching (PSM) [6] is the most widely used control group selection method. It is widespread in healthcare analyses [7–9], and is gaining ground in social sciences [10–12] and economics [13–15]. PSM matches the individuals of the case and control groups based on their *propensity score* values, which is the probability of the group (e.g. treatment) assignment conditional on the observed baseline covariates. From a practical viewpoint, various PSM methods exist [16–20] that may differ in terms of selection methodology, the ratio of the treated and untreated individuals or the nature of the selection process. On one hand, individuals can be selected into the control group with or without the replacement of the candidates. A general tendency is to apply PSM with replacement when the population from which the control group is selected is too small. Otherwise, matching without replacement can also be used. On the other hand, the ratio of the case and control groups can also be varied. 1:1 matching is common practice, but in the case of large datasets, other implementations e.g 1:M matching can also be used. Thirdly, the variety of the propensity score-based matching methods also increases by the fact that during the selection of the individuals, greedy or optimal matching can also be applied. In the first case, untreated subject whose propensity score is the closest to the score of a given treated subject is selected and matched. When optimal matching is used, the aim is to minimize the total within-pair difference of the propensity scores, and the pairing is optimized globally [21, 22]. Although, in the literature, different extensions (e.g. radius matching, kernel matching, inverse probability weighting, GPS-CDF method, etc. [23–26]) of the basic PSM methods were also proposed, in everyday practice the greedy 1:1 matching without replacement is the most widely used form of the PSM method.

Despite the popularity of these methods, they have also received much criticism [27–29]. Most of the critical comments point to the possible imbalance between the case group and the control group. It was shown in a recent article [30], that out of 1000 articles using PSM (published between 1983 and 2015), only 6% used any iterative balance checking procedure. In the remaining 94% of the articles, simple 1:1 greedy PSM was applied without any balance checking. King and Nielsen highlighted that PSM is blind to the often large imbalance that can be eliminated by approximating full blocking with other matching methods [30]. Moreover, they pointed out that propensity score matching may increase imbalance even relative to the original data. In a review article published by P. Austin [31] some articles were also identified containing imbalance on the baseline covariates between the case and control groups despite the suitable application of the PSM method. In [32] cardiovascular studies were examined, and authors showed that propensity score methods are not necessarily superior to conventional covariate adjustment.

The main problem with the most widely applied form of the PSM method presumably originates from the application of dimension reduction of the original feature space. Namely, pairing is performed in the 1-dimensional space of the propensity score values, which reduced space might hide the distributions of the original dimensions (features). Covariates that equally affect the probability of individuals belonging to the treated (or diseased) group also affect the value of the propensity score to the same extent. However, the distribution of these variables may be different, and this difference will no longer appear in the 1-dimensional probability values. As long as the matching is performed in the 1-dimensional space of the propensity scores, these differences can not be taken into account during the matching procedure. Publications [33] and [34] also highlighted that matched-pair analysis has to be performed only when matched individuals are highly correlated, but matching subjects having similar propensity scores do not necessarily result in matched subjects with similar covariate values. Based on these considerations, we regard matching in the original *n*-dimensional vector space or its subspace more suitable than in the 1-dimensional space of propensity scores. The mentioned subspace refers to the covariates which should be included in the propensity score model. Based on recommendations of Austin [35] and Brookhart [36], all variables that affect both the exposure of the group membership and the outcome of the study should always be taken into account.

In this paper, a novel weighted *k*-nearest neighbour matching method is proposed to select the most suitable individuals into the control group. The proposed method and the most widely used PSM are similar as both methods calculate the probabilities of group membership (e.g. treatment assignment) using a logistic regression (LR) fit. However, PSM pairs the individuals in the reduced 1-dimensional space of propensity scores, the proposed method performs the matching process in the original feature space of covariates utilizing the regression coefficients of the LR model as weight factors of the dimensions. In this way, on the one hand, the proposed method eliminates the main disadvantage of the PSM method, but at the same time retains its advantage by utilizing its regression coefficients as weight factors of the covariates. Monte Carlo simulations show, that the proposed method can overcome the aforementioned shortcomings of the PSM method and may result in a more balanced control group.

In the following, the propensity score matching method and the proposed Weighted Nearest Neighbours Control Group Selection with Error Minimization method (WNNEM) are introduced. Following this, a Monte Carlo study is presented to compare the most widely applied form of the PSM method with the WNNEM method and to highlight the main advantages of the proposed method. To demonstrate the relative utility of the proposed method, some head to head comparisons with other well-known methods (stratified matching, nearest neighbour matching and Mahalanobis metric matching) were also included in our study.

## Methods

Matching-based control group selection methods aim to select and pair individuals from a set of potential candidates (*X*_*C*_) to individuals of the case group (*X*_*T*_). Individuals **X**_*i*_ ∈ {*X*_*C*_ ∪ *X*_*T*_} are characterized by *n* (n∈N) descriptive features (e.g. age, gender, diagnoses) denoted as *f*_1_, *f*_2_, …, *f*_*n*_. Therefore, each subject is denoted as an $X_{i}=\left[x_{if_{1}},x_{if_{2}},\ldots ,x_{if_{n}}\right]$ vector of variables, where *i* = 1, 2, …, *L* and *L* = |*X*_*C*_ ∪ *X*_*T*_|. The aim of control group selection methods is to select such an *X*_*UT*_ ⊂ *X*_*C*_ control group, that is balanced to the case group, meaning that the distributions of the variables in both sets are similar. Naturally, *X*_*T*_ and *X*_*UT*_ must be disjoint sets, that is *X*_*T*_ ∩ *X*_*UT*_ = ∅. To ensure this requirement, *X*_*T*_ and *X*_*C*_ must also be disjoint (*X*_*T*_ ∩ *X*_*C*_ = ∅).

### Propensity score matching

Propensity score matching refers to matching techniques that are based on propensity scores (PS). Propensity score is the conditional probability of treatment assignment based on the observed baseline covariates:

$$\begin{array}{c} p_{i}=Pr\left(z_{i}=1|X_{i}\right), \end{array}$$ (1)

where *p*_*i*_ denotes the propensity score for the *i*-th participant, and *z*_*i*_ ∈ {0, 1} denotes the treatment variable in such a way that *z*_*i*_ = 0 refers to the control (e.g untreated) group and *z*_*i*_ = 1 refers to the case (e.g. treated) group. Subjects characterized by the same properties have the same propensity scores.

In retrospective observational studies, the true propensity score is unknown and has to be estimated from available data. Usually, it is estimated using a logistic regression model, but other methods have also been examined and used (e.g. recursive partitioning [37], random forests [38], bagging and boosting [39, 40] and neural networks [35, 41]). When the dependent variable is dichotomous, logistic regression is the most commonly used method to estimate the propensity scores. In this case, treatment status is regressed on the observed baseline covariates and propensity scores are estimated by the fitted model. The multiple linear regression function estimated by the logistic regression model can be defined as follows:

$$\begin{array}{c} logit\left(p\right)=b_{0}+b_{1}f_{1}+b_{2}f_{2}+\cdots +b_{n}f_{n} \end{array}$$ (2)

where

$$\begin{array}{c} logit\left(p\right)=ln(\frac{p}{1-p}) \end{array}$$ (3)

and *p* is the probability of being exposed. Furthermore, the values *b*_*k*_ (*k* = 1, 2, …, *n*) are the regression coefficients that describe the relative effects of the covariates (*f*_*k*_) on the status of group (e.g. treatment) assignment. The propensity score estimated by logistic regression is calculated as:

$$\begin{array}{c} p=\frac{e^{\left(b_{0}+b_{1}f_{1}+b_{2}f_{2}+\cdots +b_{n}f_{n}\right)}}{1+e^{\left(b_{0}+b_{1}f_{1}+b_{2}f_{2}+\cdots +b_{n}f_{n}\right)}} \end{array}$$ (4)

PSM methods essentially follow the nearest neighbour-based approach. That is, that individual is selected from the candidates pairing whose propensity score is the most similar to the propensity score of the individual to be paired in the case group. If multiple subjects from the candidates have equally close propensity scores to the propensity score of the sample subject, one of those is selected at random. As the greedy method does not contain any restrictions concerning the maximum acceptable difference between the propensity scores of the two matched subjects, practical implementations often take into account a threshold parameter for the selection [42]. Individuals within a certain distance of the propensity scores (*caliper size*) are matched together, and subjects that fall outside this caliper are neglected. Various suggestions have been made for optimal caliper size in the literature [42–44], but usually 0.2 of the standard deviation of the logit of the propensity scores is recommended [42].

### Weighted nearest neighbours control group selection with error minimization

As it was described in the Introduction, PSM methods are constantly in the midst of criticism due to the imbalance of the covariates observed in some studies. The disadvantage of the most widely used PSM and all other propensity score-based methods is that they perform the pairing of individuals in the 1-dimensional space of the propensity scores. Additionally, uncertainty is also increased by the fact that propensity scores are estimated and not known a priori. Although earlier some methods have been proposed to pair individuals in the original vector space of the features [45–47], to the best of our knowledge none of them utilizes the result of fitting a logistic regression model during the matching performed in the original vector space. In this section, such a novel weighted nearest neighbours-based control group selection method is proposed in which the relevance of the covariates is estimated by fitting a logistic regression model, furthermore control group selection is performed in the original vector space of the covariates by calculating the weighted distances of the treated individuals and the possible candidates.

The suggested Weighted Nearest Neighbours Control Group Selection with Error Minimization method (WNNEM) considers each subject as an *n*-dimensional data point in an *n*-dimensional space, where each covariate (*f*_*k*_, *k* = 1, …, *n*) represents a unique dimension. This way, the problem of control group selection can be interpreted as a distance minimization problem. To select a proper control group, we have to identify such individuals from the candidates that lie close to the individuals of the case group. The concept of lying close can be defined in numerous ways. In the case of the proposed method, multivariate matching is performed in which the adjusted odds ratio (OR) values of the fitted multivariable logistic regression model are utilized as weighting factors of the covariates to compute the distances between the individuals.

Before the whole algorithm is presented, two aspects have to be clarified. Firstly, the term distance and secondly the suggested weighting method has to be specified.

Generally, as individuals may be characterized by different types of variables (binary, nominal, ordinal, numerical), the distance calculation method to be applied must be able to handle different data types. Furthermore, as the significance of the covariates may differ, distances have to be calculated separately for each dimension. The third requirement of distance calculation is that the dissimilarity measures with identical values have to express the same degree of dissimilarity.

To fulfil these requirements, the proposed algorithm calculates the differences for each dimension separately and converts all dissimilarity values into the range of [0, 1]. The distance calculation for different data types is performed as follows:

- In the case of binary variables, the simple matching distance is calculated. That is,
  $$\begin{array}{c} d_{ij}^{\left(f\right)}=\{\begin{array}{cc} 0 & \text{if}\;x_{if}=x_{jf}\\ 1 & \text{if}\;otherwise \end{array} \end{array}$$ (5)
  where $d_{ij}^{\left(f\right)}$ yields the distance of individuals **X**_*i*_ ∈ *X*_*T*_ and **X**_*j*_ ∈ *X*_*C*_, *x*_*if*_ is the value of individual **X**_*i*_ on binary variable *f*, and *x*_*jf*_ of **X**_*j*_, respectively.
- In the case of nominal variables, either the simple matching distance presented before (Eq 5) can be calculated or these variables can be coded as a set of binary variables and the distance can be calculated as the normalized distance of binary features, where the normalization constant represents the number of possible values of the nominal variable.
- In the case of numerical variables, the dissimilarity measure can be calculated as the difference of the original values. As the distance calculated in this way depends on the range of the original values, normalization is needed to achieve uniform significance for the same dissimilarities and to make them comparable to the dissimilarity measures calculated on other types of attributes. To fulfil this requirement, min-max normalization must be performed separately for each numerical dimension to map the original values into the range of [0, 1] as follows:
  $$\begin{array}{c} x_{if}^{*}=\frac{x_{if}-min_{f}}{max_{f}-min_{f}} \end{array}$$ (6)
  where *x*_*if*_ denotes the original value of individual **X**_*i*_ in the *f*-th dimension without normalization, *min*_*f*_ represent the minimum and *max*_*f*_ the maximum value measured in the *f*-th dimension taking into account all individuals from *X*_*T*_ ∪ *X*_*C*_, and $x_{if}^{*}$ yields the normalized value of the individual **X**_*i*_ with regard to the *f*-th covariate. Subsequently, the distance of individuals **X**_*i*_ ∈ *X*_*T*_ and **X**_*j*_ ∈ *X*_*C*_ is calculated as the differences of their normalized feature values:
  $$\begin{array}{c} d_{ij}^{\left(f\right)}=\left|x_{if}^{*}-x_{jf}^{*}\right| \end{array}$$ (7)
- In the case of ordinal variables, the ordered values have to be coded as ranks and the distance can be calculated as the aforementioned distance of numerical values.

After ensuring that meaning of the dissimilarity values is identical in each dimension, the next step is to weight them according to their relevance to treatment assignment. Previously mentioned, the adjusted odds ratio values of the multivariable logistic regression model fitted on the status of treatment assignment are utilized for this purpose. Generally, the odds ratio is the probability of a characteristic being present divided by the probability of the same characteristic being absent. In our case, the adjusted odds ratio for each independent variable can be obtained by applying the exponential function to the corresponding regression coefficient (*b*_*k*_) obtained from the multivariable logistic regression model (Eq 2). That is, adjusted odds ratios as the weights of the covariates are calculated as follows:

$$\begin{array}{c} w_{k}=OR_{k}=e^{b_{k}} \end{array}$$ (8)

where *w*_*k*_ denotes the weighting factor of the *k*-th covariate (*k* = 1, 2, …, *n*).

The proposed WNNEM method calculates the distances for individuals **X**_*i*_ ∈ *X*_*T*_ and **X**_*j*_ ∈ *X*_*C*_ the following way:

$$\begin{array}{c} dist\left(X_{i},X_{j}\right)=\sum_{k=1}^{n}w_{k}d_{ij}^{\left(k\right)} \end{array}$$ (9)

where $d_{ij}^{\left(k\right)}$ represents the normalized dissimilarity value of **X**_*i*_ and **X**_*j*_ in the *k*-th dimension, and *w*_*k*_ is the weighting factor of dimension *k*.

The presented weighted attribute distance is utilized to match the *best pairs* of candidates (*X*_*C*_) and individuals of the treated group (*X*_*T*_). Basically, the *best pair* for each **X**_*i*_ ∈ *X*_*T*_ is that **X**_*j*_ ∈ *X*_*C*_ for which *dist*(**X**_*i*_, **X**_*j*_) is minimal. This way, the matching procedure can be regarded an optimization problem, where ∑_*i*,*j*_ *dist*(**X**_*i*_, **X**_*j*_) has to be minimized.

Our practical experiments show that for 1:1 matching, an adequate solution can be found even without the use of a complex optimization algorithm. The only problem that needs to be handled during optimization is management of the pairing process of those candidates which lie closest to more than one individual from the case group. These candidates are called *candidates in conflict* and formally are defined as follows: **X**_*j*_ ∈ *X*_*C*_ is a *candidate in conflict* if *d*(**X**_*i*_, **X**_*j*_) is minimal for more than one **X**_*i*_ ∈ *X*_*T*_.

For handling these conflicts, the order of the neighbours has to be determined. Let *NN*_1_(**X**_*i*_) denote the closest and *NN*_2_(**X**_*i*_) the second closest neighbour to individual **X**_*i*_ ∈ *X*_*T*_. By definition, *NN*_1_(**X**_*i*_) and *NN*_2_(**X**_*i*_) are calculated as follows:

$$\begin{array}{c} NN_{1}\left(X_{i}\right)=\underset{X_{j}\in X_{C}}{\mathrm{argmin}}\left(dist\left(X_{i},X_{j}\right)\right) \end{array}$$ (10)

$$\begin{array}{c} NN_{2}\left(X_{i}\right)=\underset{X_{j}\in X_{C}-\left\{NN_{1}\left(X_{i}\right)\right\}}{\mathrm{argmin}}\left(dist\left(X_{i},X_{j}\right)\right) \end{array}$$ (11)

The design of the conflict-handling method to solve the competition of two individuals was inspired by the Vogel-Korda method: instead of a greedy selection, the second neighbours of the treated individuals are also taken into account. That is, the candidate in conflict is matched to the individual for which the error function is greater. The error function is calculated as the distance of the first and second neighbours of the individuals as follows:

$$\begin{array}{c} E\left(X_{i}\right)=\left|dist\left(X_{i},NN_{1}\left(X_{i}\right)\right)-dist\left(X_{i},NN_{2}\left(X_{i}\right)\right)\right| \end{array}$$ (12)

In this way, the problem of two competing individuals, **X**_*l*_ and **X**_*m*_ ∈ *X*_*T*_, is solved. In the case of multiple competing individuals, conflicts are handled by dynamic programming. First, the conflict with the largest error is resolved followed by the others in descending order. This principle is applied iteratively until all the conflicts are resolved.

In summary, the steps of the proposed Weighted Nearest Neighbours Control Group Selection with Error Minimization method (WNNEM) are presented by Algorithm 1. It should be noted that although the presented WNNEM algorithm performs 1:1 matching, it can be easily extended for 1:M matching as well. In this case, in Step 3 the set of unpaired elements (*X*_*unpaired*_) has to be defined in the way, that each individual of the case group (*X*_*T*_) should be placed *M* times into the set *X*_*unpaired*_. The other parts of the algorithm do not change.

**Algorithm 1:** Weighted Nearest Neighbours Control Group Selection with Error Minimization (WNNEM)

**Input:** *X*_*T*_ case group, *X*_*C*_ set of candidate individuals

**Output:** *X*_*UT*_ control group

**1** Perform a logistic regression to estimate *w*_*k*_ weights for all covariates.

**2** Normalize *X*_*T*_ and *X*_*C*_ collectively using feature scaling and calculate the **D** = *dist*(**X**_*i*_, **X**_*j*_) distance matrix for all pairs of individuals of **X**_*i*_ ∈ *X*_*T*_ and **X**_*j*_ ∈ *X*_*C*_ by Eq 9.

**3** Set

  *X*_*unpaired*_ = *X*_*T*_

  *X*_*UT*_ = ∅

**4** Determine *NN*_1_(**X**_*i*_) and *NN*_2_(**X**_*i*_) based on the distance matrix **D** for all **X**_*i*_ ∈ *X*_*unpaired*_.

**5** Calculate *E*(**X**_*i*_) for all **X**_*i*_ ∈ *X*_*unpaired*_ by Eq 12.

**6** For all *i* ∈ {*arg*(*X*_*unpaired*_)}

  Set $l={\mathrm{argmax}}_{X_{i}\in X_{unpaired}}\left(E\left(X_{i}\right)\right)$

  If *NN*_1_(**X**_*l*_)∉*X*_*UT*_:

   *X*_*UT*_ = *X*_*UT*_ ∪ {*NN*_1_(**X**_*l*_)}

   *X*_*unpaired*_ = *X*_*unpaired*_ − {**X**_*l*_}

   Set *m* = *arg*(*NN*_1_(**X**_*l*_))

   Set **D**(*i*, *m*) = ∞ for all *i* ∈ {*arg*(*X*_*T*_)}

**7** Repeat *Steps 4 to 6*, till *X*_*unpaired*_ ≠ ∅.

### Comparison of the PSM and WNNEM methods—Monte Carlo study

#### Study design

To present the effectiveness of the proposed method, a Monte Carlo simulation-based experimental study was performed. In this study, the quality of the control group arising from the proposed WNNEM method was compared to the quality of the control groups arising from the following matching methods: (i) two greedy PSM methods, (ii) nearest neighbour matching (NNM) [48], (iii) Mahalanobis metric matching (MMM) [47] and (iv) stratified matching (SM) [49]. In the cases using PSM methods, the most widely applied form, namely, the greedy 1:1 propensity score matching performed without replacement of individuals was applied. The only difference between the two forms of the PSM methods applied was the ‘caliper size’ setting. In the first case (PSM_02), the caliper size was set as 0.2 of the standard deviation of the logit of the propensity scores. In the second case (PSM_DYN), the caliper size in each simulation was determined dynamically and was set at the minimal value for which 1:1 matching could be performed.

As the greedy nature of the PSM, the nearest neighbour matching and the Mahalanobis metric matching makes them sensitive to the order of the candidates, these methods were run 10 times on each generated dataset in such a way, that the matching order was randomized in each experiment. In contrast, when the WNNEM method or stratified matching was applied, the control group selection was performed only once for each of the generated datasets, because of the deterministic nature of these algorithms.

#### Datasets

For the comparisons, three scenarios of datasets with varying feature characteristics were designed. For each scenario, 100 datasets were generated randomly with the same distribution parameters predefined for the covariates. As a result, each scenario contained 100 different datasets with the same number of individuals. That is, in total, we evaluated the accuracy of the various control group selection methods on 300 datasets. Since the PSM methods, the nearest neighbour matching and the Mahalanobis metric matching ran 10 times on each dataset, the presented research is based on a total of 12600 outcome evaluations.

*Scenario I* In the first scenario, datasets contained 1000 individuals and correspond to the dataset widely applied in theoretical PSM studies [50]. Individuals were characterized by 10 binary variables, each from a Bernoulli distribution (*x*_*j*_ ∼ B(0.5), *j* = 1, …, 10).

The calculation of the probability of treatment assignment was based on the following logistic regression model:

$$\begin{array}{c} \begin{array}{cc} \text{logit}(p_{i,treat}) & =b_{0,treat}+\\ & b_{L}x_{i1}+b_{L}x_{i2}+b_{L}x_{i3}+b_{M}x_{i4}+b_{M}x_{i5}+\\ & b_{M}x_{i6}+b_{H}x_{i7}+b_{H}x_{i8}+b_{VH}x_{i9}+b_{VH}x_{i10} \end{array} \end{array}$$ (13)

where weights *b* denote low (L), medium (M), high (H) or very high (VH) effect on treatment assignment.

For each subject, a treatment status indicator (*Z*_*i*_) was generated from a Bernoulli distribution with a subject-specified probability equal to *p*_*i*,*treat*_ (*Z*_*i*_ ∼ B(*p*_*i*,*treat*_)). The treated group consisted of subjects where *Z*_*i*_ = 1, while subjects where *Z*_*i*_ = 0 were assigned to the untreated group (from which the control group was selected). The weights (*b*_*L*_, *b*_*M*_, *b*_*H*_ and *b*_*VH*_) were assigned in such a way that approximately 25% of the subjects were treated. The applied weight coefficients were as follows:

- correction for binary: *b*_0,*treat*_ = −1.344090
- low: *b*_*L*_ = log(1.1)
- medium: *b*_*M*_ = log(1.25)
- high: *b*_*H*_ = log(1.5)
- very high: *b*_*VH*_ = log(2.1)

*Scenario II* The second scenario models such studies in which fewer descriptive variables are available. In this scenario, each individual was characterized by 1 ordinal and 5 binary variables. The ordinal variable represents 5 age groups, while the binary variables may represent, for example, the gender of the subject or various diagnoses. In this scenario, 700 individuals were simulated in each dataset, and the ratio of the candidate subjects to the treated individuals in the 100 datasets was between 2.0 and 3.1.

The assignment of weights to the descriptive variables was as follows: the ordinal variable (*x*_1_) has very high effect, the binary variables have low (*x*_2_), medium (*x*_3_ − *x*_5_) and high (*x*_6_) effect on the status of treatment (Eq 14).

$$\begin{array}{c} \begin{array}{cc} \text{logit}(p_{i,treat}) & =b_{0,treat}+\\ & b_{VH}x_{i1}+b_{L}x_{i2}+b_{M}x_{i3}+b_{M}x_{i4}+b_{M}x_{i5}+\\ & b_{H}x_{i6} \end{array} \end{array}$$ (14)

The applied weight coefficients, in this case, were the same as in Scenario I.

*Scenario III* The third scenario is similar to the second one regarding the attributes of individuals and the total number of subjects in each dataset. However, it simulates a more difficult control group selection problem. Although each dataset still contained 700 individuals, the number of treated individuals in the case of the third scenario is higher than in the second one. While in scenario II, the size of the treated group varied between 24.5 and 33.0 percent of the dataset, in the case of the third scenario, it is between 31.7 and 40.7 percent. In other words, the third scenario simulates a more difficult case, where the ratio of the candidate individuals to the treated subjects is lower (*X*_*C*_/*X*_*T*_ ≈ [1.5, 2.2]) than in the second scenario (*X*_*C*_/*X*_*T*_ ≈ [2.0, 3.1]). Therefore, in this scenario, it is harder to find a proper pair for each treated individual.

To achieve a higher treatment rate, the weight coefficients were modified as follows:

- correction for binary: *b*_0,*treat*_ = −1.344090
- low: *b*_*L*_ = log(1.35)
- medium: *b*_*M*_ = log(1.6)
- high: *b*_*H*_ = log(2.1)
- very high: *b*_*VH*_ = log(3.1)

#### Methods to evaluate the similarity of the case and control groups

To validate the result of control group selection, the similarity of the case group and the control group has to be evaluated. In our study, comparison was conducted the following ways: (1) comparing the distributions of the covariates and (2) measuring the similarity of the matched pairs.

In the first case, two different performance evaluations were done. On the one hand, the most common balance metric for comparing propensity score methods, namely the standardized mean difference (SMD) [51] was calculated. Additionally, the most commonly applied goodness of fit tests were also calculated. In the case of continuous covariates with normal distribution, Student two-sample *t*-test [52] was applied, and in the case of non-normal continuous covariates the Kolmogorov-Smirnov test [53, 54] was used. In the case of testing the similarity of categorical variables, Chi-square test [55] was applied.

The most common drawback of the previously mentioned methods is that they only examine the similarity of the values of a single covariate. As the Hansen and Bowers imbalance test [56] is applied for more complex evaluation in biomedical studies, this aggregated imbalance measure was also calculated in this study. The main virtue of this measure is that it is an aggregated imbalance test, which allows the imbalance of all covariates to be evaluated simultaneously. The application limit of the test, namely that it can be applied for 1:1 matching without replacement, made it ideal for our study. To extend the complex evaluation for all covariate distributions together, the Distribution Dissimilarity Index (DDI) presented in [57] was also utilized. This measure first compares the disparity between histograms for each covariate separately and calculates the differences of the frequencies of the histogram bins before the differences are totalled and normalized.

As the similarity of the distributions of the covariates does not mean that the paired individuals are also similar, two aggregated pairwise similarity measures, the Nearest Neighbour Index (NNI) and the Global Dissimilarity Index (GDI) [57], were also calculated for each comparison. The common characteristic of these measures is that the overall distance of the case and control groups is calculated by evaluating the pairwise distances of the matched subjects. The NNI examines for each treated subject whether the paired individual is the closest individual in the given population. The GDI evaluates the similarity of the paired elements more precisely. First, it calculates the normalized distances of the paired subjects, then these distances are totalled and normalized for all matched pairs. In this way, the Global Dissimilarity Index expresses the magnitude of the distances of the paired individuals as well.

## Results

In this section, the results of the study are presented. For a fair comparison, not only two types of the PSM method were compared to the proposed WNNEM method, but other distance-based approaches (nearest neighbour matching, Mahalanobis metric matching) and the widely used stratified matching were also examined.

### Scenario I

As mentioned before, in this scenario each dataset contains 1000 individuals, each characterized by 10 binary covariates.

Table 1 shows the mean value of the NNI, GDI and DDI distance measures with their standard deviations. All presented dissimilarity measures may fall within the range of [0, 1], and the value of zero expresses that the case and control groups are identical. Consequently, the greater the dissimilarity value, the higher the difference of the case and control groups is. The results show that the WNNEM method performed the best in terms of control group selection. All dissimilarity values, both for the paired evaluations (NNI and GDI) and the distribution-based evaluation (DDI), are lower in the case of control groups selected by the WNNEM method than by the other methods.

**Table 1:** Dissimilarity measures for Scenario I. The Nearest Neighbour Index (NNI) and the Global Dissimilarity Index (GDI) present the results of the evaluation of the similarity of matched pairs. The Distribution Dissimilarity Index (DDI) evaluates the similarity of the histograms of covariates.

	NNI	GDI	DDI
WNNEM	0.0599±0.0031	0.0613±0.0046	0.0120±0.0033
SM	0.5737±0.0248	0.5737±0.0248	0.5737±0.0248
NNM	0.0649±0.0034	0.0747±0.0057	0.0152±0.0040
MMM	0.0661±0.0036	0.0760±0.0064	0.0150±0.0041
PSM_02	0.3252±0.0290	0.3588±0.0339	0.0664±0.0179
PSM_DYN	0.2875±0.0259	0.3237±0.0327	0.0160±0.0038

To evaluate the results of the selected control groups, the similarities of the covariates were also evaluated separately. Firstly, the SMD values were calculated for all covariates and for all matching methods applied. The detailed results are presented in Table 2. It can be seen that all matching methods resulted in well-balanced control groups as all SMD values are less than 0.1. There are only small differences between the values in favour of the distance-based methods. On covariate *x*_1_ the nearest neighbour matching method, on covariates *x*_2_ and *x*_3_ the Mahalanobis metric matching and on covariates *x*_4_ − *x*_10_ the WNNEM method resulted in the most balanced control groups. Given that covariates *x*_1_ − *x*_3_ were associated only with low weights to the group membership (Eq 13), we can say that the WNNEM method gave the most accurate balancing on the major covariates.

**Table 2:** SMD values for Scenario I. The table shows the average value of the standardized mean differences and their standard deviations for each covariate separately.

	*x*_1_	*x*_2_	*x*_3_	*x*_4_	*x*_5_
WNNEM	0.034±0.030	0.029±0.027	0.025±0.024	0.022±0.020	0.026±0.021
SM	0.057±0.046	0.057±0.042	0.044±0.036	0.057±0.047	0.057±0.039
NNM	0.024±0.019	0.030±0.022	0.026±0.021	0.028±0.021	0.027±0.021
MMM	0.027±0.026	0.024±0.022	0.023±0.019	0.030±0.024	0.031±0.030
PSM_02	0.039±0.029	0.035±0.024	0.035±0.029	0.033±0.027	0.031±0.024
PSM_DYN	0.034±0.024	0.031±0.023	0.032±0.024	0.028±0.021	0.031±0.023
	*x*_6_	*x*_7_	*x*_8_	*x*_9_	*x*_10_
WNNEM	0.023±0.019	0.021±0.018	0.020±0.016	0.022±0.016	0.018±0.014
SM	0.056±0.040	0.059±0.046	0.048±0.032	0.059±0.043	0.065±0.050
NNM	0.027±0.019	0.034±0.027	0.030±0.024	0.033±0.022	0.045±0.030
MMM	0.026±0.024	0.032±0.031	0.027±0.026	0.035±0.033	0.046±0.037
PSM_02	0.035±0.024	0.037±0.027	0.034±0.023	0.036±0.025	0.042±0.029
PSM_DYN	0.029±0.021	0.031±0.025	0.029±0.020	0.028±0.020	0.030±0.022

**Fig 1:**
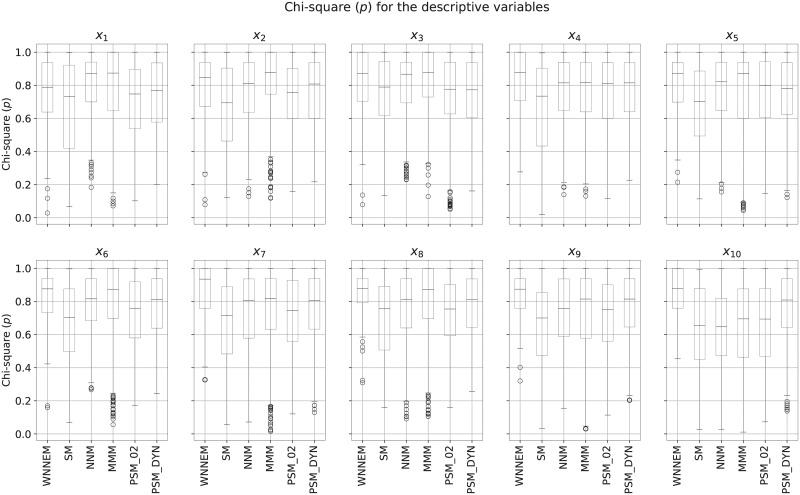
Distribution of covariates in Scenario I. Distribution of the Chi-square *p*-values calculated for each covariate based on all simulations.

In the next step, the similarity of the covariates for the case and control groups was tested by the Chi-square test. A higher *p*-value means a more balanced control group in terms of a given covariate. The detailed results are presented as box plots in Fig 1. As can be seen, the median of the *p*-values for covariates *x*_4_ − *x*_10_ is the highest in the case of WNNEM method, and for covariates *x*_5_ − *x*_10_ the interquartile ranges of the WNNEM method is the smallest. Furthermore, the first quartiles from *x*_4_ to *x*_10_ are also the highest in the case of the WNNEM method.

**Fig 2:**
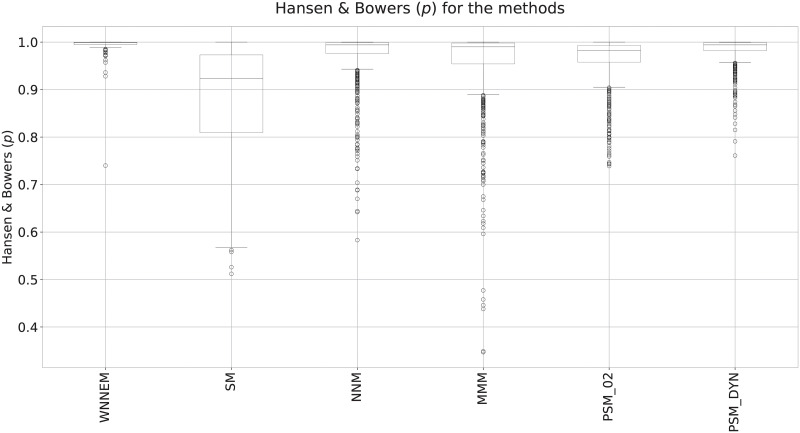
Results of Hansen and Bowers tests in Scenario I. Comparison of the *p*-values of the Hansen and Bowers test for the WNNEM method, the stratified matching, the nearest neighbour matching, the Mahalanobis metric matching and two variants of the PSM method.

To summarize the balances of the covariates, the widely used Hansen and Bowers test was also evaluated for all matching algorithms. In the Hansen and Bowers test, covariates are considered poorly balanced if the test value is significant (*p* < 0.05). The higher the *p*-value, the more similar the case and control groups are. The *p*-values for all simulations are presented as box plots in Fig 2.

Fig 2 clearly shows that the WNNEM method resulted in the most balanced control groups in terms of the Hansen and Bowers test. Not only the interquartile range is the smallest in the case of the WNNEM method, but the tail of the box as well. Furthermore, only a few outlier simulations can be observed, and, except for one simulation, they all have high *p*-values. All these facts support the advantages of applying the proposed WNNEM method.

### Scenario II

The second scenario simulates such studies in which the age of the patients and another 5 binary parameters are considered as covariates.

The overall statistics of the control group selections are presented in Table 3. It can be seen that the proposed WNNEM method performed better in terms of GDI and DDI measures than the other methods. However, in terms of the Nearest Neighbour Index, the lowest value can be seen at the nearest neighbour matching, but the difference is negligible (0.005). Furthermore, we can observe that distance-based algorithms generally give 1 order of magnitude better results than PSM methods, except the DDI value of the PSM_DYN method. Furthermore, by comparing these results to the results of Scenario I, it can be seen, that in this case, the selected control groups are more similar to the case group.

**Table 3:** Dissimilarity measures for Scenario II. The Nearest Neighbour Index (NNI) and the Global Dissimilarity Index (GDI) present the results of the evaluation of the similarity of matched pairs. The Distribution Dissimilarity Index (DDI) evaluates the similarity of the histograms of covariates.

	NNI	GDI	DDI
WNNEM	0.0073±0.0040	0.0069±0.0041	0.0036±0.0028
SM	0.0382±0.0190	0.0382±0.0190	0.0382±0.0190
NNM	0.0065±0.0033	0.0077±0.0040	0.0046±0.0023
MMM	0.0068±0.0035	0.0082±0.0044	0.0041±0.0027
PSM_02	0.0303±0.0169	0.0312±0.0174	0.0251±0.0148
PSM_DYN	0.0162±0.0104	0.0178±0.0119	0.0088±0.0052

**Fig 3:**
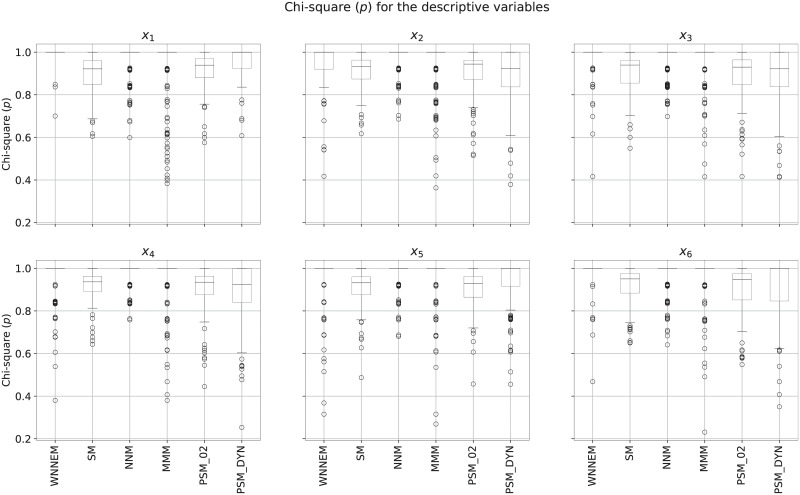
Distribution of covariates in Scenario II. Distribution of the Chi-square *p*-values calculated for each covariate based on all simulations.

For further evaluation, the similarity of the covariates was also calculated. Evaluating the SMD measures for the covariates separately, we found that for all covariates and for all methods the average values are below 0.1, so the matched control groups are well balanced on each covariate. The similarity of the case and control groups measured by the Chi-square test is presented in Fig 3. It is important to emphasize that in the case of completely missing boxes, the first, second and third quartiles of the *p*-values were all equal to 1. In the case of partially missing boxes, the median was equal to 1, therefore the third quartile and maximum value were equal. Consistent with the results observed for the NNI, GDI and DDI indices, Fig 3 also emphasizes the better performance of distance-based measures (WNNEM, nearest neighbour matching and Mahalanobis metric matching) for this data set.

**Fig 4:**
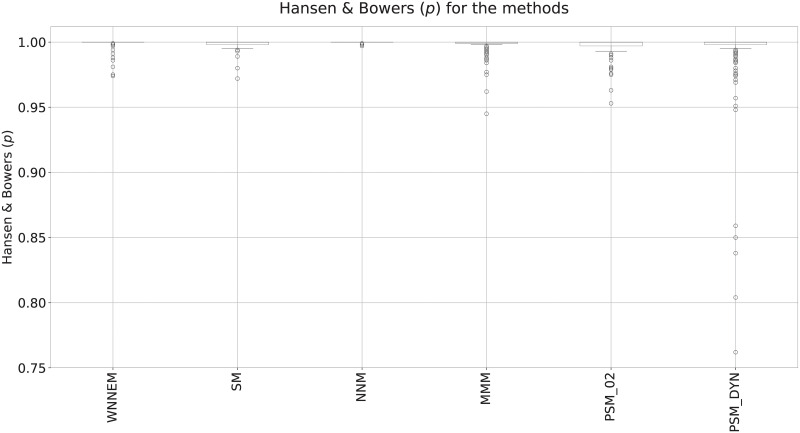
Results of Hansen and Bowers tests in Scenario II. Comparison of the *p*-values of the Hansen and Bowers test for the WNNEM method, the stratified matching, the nearest neighbour matching, the Mahalanobis metric matching and two variants of the PSM method.

Evaluating the Hansen and Bowers test (Fig 4), we can establish that all methods have selected very similar control groups to the case groups. The differences between the boxes are marginal, but the PSM method with a dynamically determined caliper size parameter resulted in less similar control groups more times.

In this example, we have seen that all methods were able to select perfectly balanced control groups. This is due to the lower variability of features of the individuals and the relatively large number of available candidates.

### Scenario III

The third scenario is similar to the second one but it simulates a more difficult control group selection problem as the ratio of the candidate individuals to the treated ones is less than in Scenario II. In this scenario, on average, only 1.7 candidate individuals were available per treated person, while in Scenario II this value was 2.5.

In Table 4 the overall dissimilarity measures are presented. By comparing Tables 3 and 4, it can be seen that in the case of the third scenario, it was harder to select fully balanced control groups. However, Table 4 shows that the distance-based methods were still able to select more balanced control groups than the greedy PSM methods, and the stratified matching resulted in the worst dissimilarity measures. The nearest neighbour matching and the Mahalanobis metric matching seem to give better results in terms of NNI and GDI measures than the WNNEM method, but the deviation is less than 0.01.

**Table 4:** Dissimilarity measures for Scenario III. The Nearest Neighbour Index (NNI) and the Global Dissimilarity Index (GDI) present the results of the evaluation of the similarity of matched pairs. The Distribution Dissimilarity Index (DDI) evaluates the similarity of the histograms of covariates.

	NNI	GDI	DDI
WNNEM	0.0304±0.0114	0.0351±0.0165	0.0115±0.0066
SM	0.1151±0.0334	0.1151±0.0334	0.1151±0.0334
NNM	0.0212±0.0065	0.0297±0.0114	0.0144±0.0058
MMM	0.0234±0.0079	0.0321±0.0129	0.0144±0.0067
PSM_02	0.1035±0.0335	0.1093±0.0362	0.0839±0.0304
PSM_DYN	0.0663±0.0255	0.0817±0.0352	0.0187±0.0086

**Fig 5:**
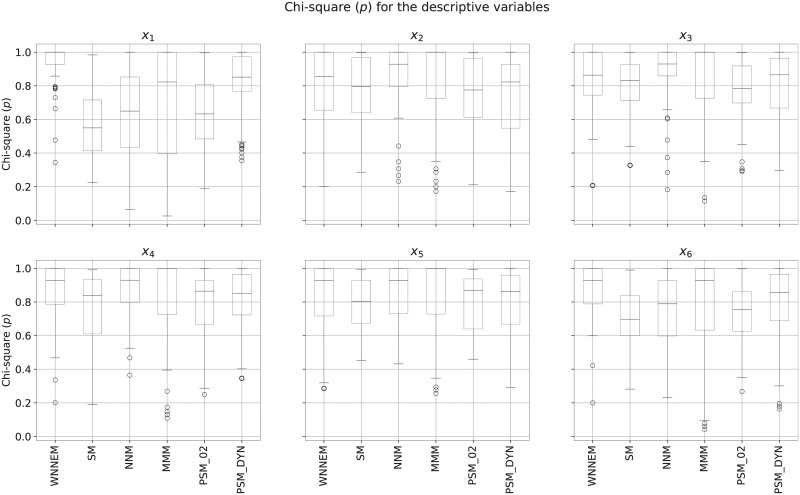
Distribution of covariates in Scenario III. Distribution of the Chi-square *p*-values calculated for each covariate based on all simulations.

Fig 5 details the covariate imbalances separately. As can be seen, the WNNEM method was able to select better-balanced control groups than the PSM methods and the stratified matching on all covariates. Comparing the WNNEM method to other distance-based methods we can see that the WNNEM method was able to achieve more balanced results on covariates with high (*x*_6_) and very high (*x*_1_) effect on the group assignment (e.g. treatment assignment). The calculated average SMD values were still below 0.1 for all methods.

**Fig 6:**
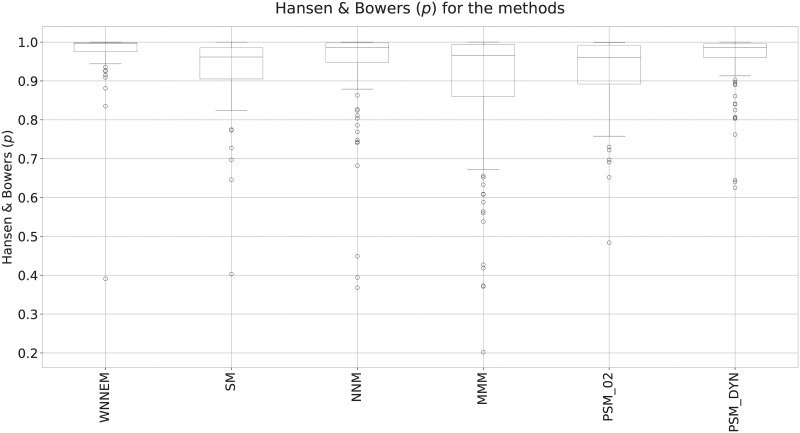
Results of Hansen and Bowers tests in Scenario III. Comparison of the *p*-values of the Hansen and Bowers test for the WNNEM method, the stratified matching, the nearest neighbour matching, the Mahalanobis metric matching and two variants of the PSM method.

Fig 6 presents the overall results of the Hansen and Bowers tests. By comparing Fig 4 and Fig 6, it can be seen that in the case of the third scenario, it was harder for each method to select a fully balanced control group. However, Fig 6 shows that the WNNEM method was the most suitable to select a balanced control group. In this way, Fig 6 confirms the ability of the WNNEM method to select better control groups in harder situations. However it should be noted, that the greedy PSM with dynamically determined caliper size also gave good results, but in a few cases the resulting control groups are not well balanced. In the case of using the WNNEM method, it happened only one time.

## Discussion

Although propensity score matching is the most widely used balancing method, many publications have drawn attention to the disadvantages of using it. Probably, the main problem arises from the fact that the selection of individuals happens in a compressed one-dimensional space of the propensity scores. In this paper, a multivariate control group selection method was proposed, which performs the control group selection in the original vector space of the covariates. The proposed Weighted Nearest Neighbours Control Group Selection with Error Minimization method takes into account the nearest neighbours of the treated individuals and tries to solve the problem of candidates in conflict. Candidates in conflict are those individuals who are the closest to more than one treated subject. Distances between the individuals of treated and untreated groups are calculated as the weighted sum of the distances of the covariates. As covariates may have different effects on treatment assignment, the proposed WNNEM method weights them according to their relevance. The weighting factors for the covariates are acquired from the logistic regression model fitted on the status of treatment assignment.

Monte Carlo simulations show that in the case of 1:1 matching, the proposed WNNEM method is able to select a more balanced control group than the most widely applied greedy forms of propensity score matching. In this article, we aimed to emphasize the fact that although the most applied forms of PSM can result in a well-balanced control group, a more similar control group can be reached with a simple nearest neighbour-based method proposed in this article. The proposed method is advantageous when individuals are characterized by fewer covariates and the search space is such small that there exist many individuals for selecting as control which are the most similar pairs of more than one treated subject. If more variables are available to characterize individuals, propensity scores describe the exposure of the individuals more precisely, so individuals in conflict may also be less prevalent. As a result, the selected control group is more balanced using the PSM method.

Furthermore, it must be emphasized that the presented form of the WNNEM method solves the problem locally and does not consider further candidates in the selection process. As a result, the proposed WNNEM method does not always select the best balanced control group. This shortcoming of the proposed method can be eliminated by applying a global optimization method, e.g. particle swarm optimization or simulated annealing. In the next step of our research, the advantages of extending the proposed method in this regard will be examined. However, it should be noted that the method proposed in this article is able to select a better control group for small datasets. Moreover, in the presented simple form, this can be achieved without specifying extra parameters. Because of its simplicity, applying the proposed method may be worthwhile, however, further investigations into this topic are necessary. The other limitation of the presented method, that it can only handle covariates with positively associated with the treatment assignment. In our next work, we plan to extend the method to negatively associated covariates as well.
